# Deltex E3 ubiquitin ligase 3 inhibits colorectal cancer cell growth and regulates cell cycle progression via upregulating E2F transcription factor 1

**DOI:** 10.1007/s11033-021-06916-7

**Published:** 2022-01-31

**Authors:** Hongli Xu, Shengnan Liang, Junjie Hu, Wentong Liu, Zhiqiang Dong, Shaozhong Wei

**Affiliations:** 1 College of Biomedicine and Health, College of Life Science and Technology, Huazhong Agricultural University, Wuhan, China; 2 Hubei Cancer Hospital, Tongji Medical College, Huazhong University of Science and Technology, Wuhan, China; 3 Colorectal Cancer Medical Research Center of Hubei, Wuhan, China; 4 Brain Research Institute, Taihe Hospital, Shiyan, China

**Keywords:** Cell cycle progression, Colorectal cancer, Cyclin D3, Deltex E3 ubiquitin ligase 3, E2F transcription factor 1

## Abstract

**Background:**

The mortality rate of colorectal cancer (CRC) remains high in developing countries. Interventions that can inhibit the proliferation of tumor cells represent promising strategies in CRC treatment. Deltex E3 ubiquitin ligase 3 (DTX3) plays an essential role in tumor development and may predict the outcome of cancer patients. This study aimed to investigate the regulatory mechanisms of DTX3 in CRC progression.

**Methods and results:**

The expression of DTX3 was significantly downregulated in CRC tissues relative to normal colorectal tissues. DTX3 overexpression inhibited, while DTX3 knockout promoted the colony-forming capacity and proliferation of CRC cells. E2F transcription factor 1 (E2F1) is a key mediator of cell cycle progression that participates in the progression, metastasis, and chemoresistance of CRC. Further analysis revealed that DTX3 regulated the transcriptional activity of E2F1 in CRC cells. The transcription by E2F1 was significantly reduced with the increase in the cellular level of DTX3, while DTX3 knockout exerted an opposite effect. DTX3 knockout also increased the expression of E2F1 target genes involved in cell cycle progression, CDC2 and Cyclin D3, while PD 0332991, an inhibitor of E2F1 transcription, inhibited the expression of both proteins.

**Conclusions:**

In conclusion, DTX3 regulated CRC cell growth via regulating E2F1 and its downstream genes. These findings support further exploration of DTX3 as a potential therapeutic target for CRC.

## Introduction

Colorectal cancer (CRC) is one of the most common malignant neoplasms with approximately two million new cases and more than eight thousand deaths annually worldwide [1]. Owing to the development of early detection methods and advances in CRC treatments, the mortality rate of CRC has been steadily decreasing in high-income countries for the past two decades [2]. However, a significant increase in CRC mortality rate is observed in transitioning countries, including China, due to growing aging population and increasing dietary fat consumption [3]. The significant health and economic burden that CRC places on patients, the health care system, and society urges the development of novel intervention strategies against this malignancy [4].

Tumorigenesis is a heterogeneous and dynamic process characterized by unchecked cell growth [5]. Accordingly, interventions that can suppress the proliferation of tumor cells represent promising strategies in anti-cancer treatment [6]. The proliferation of eukaryotic cells, including tumor cells, is primarily regulated by cell cycle progression [7, 8]. The accumulation of genetic and epigenetic mutations in cells drives tumor growth by preventing cell cycle arrest, accelerating cell division, and inhibiting programmed cell death [9]. Thus, the identification of aberrantly expressed genes in CRC cells is of great clinical significance for the development of therapeutic approaches targeting uncontrolled cell growth.

Deltex E3 ubiquitin ligase 3 (DTX3), a member of the deltex family, plays an essential role in tumor development and the survival outcome of cancer patients. The *DTX3* gene was specifically amplified in patients with highly proliferative luminal breast tumors and correlated with a poor prognosis [10]. Wang et al. identified DTX3 as an oncoprotein in ovarian cancer [11]. However, a recent study reported the anti-tumor effect of DTX3 in esophageal carcinoma by suppressing the proliferation and migration of tumor cells [12]. Whether DTX3 would regulate CRC progression remains unknown.

In this study, we aimed to investigate the regulatory role of DTX3 in CRC. Firstly, the expression of DTX3 in CRC and normal colorectal tissues was compared. Then the effects of DTX3 knockout (KO) or overexpression on the proliferation and colony-forming capacity of CRC cells were investigated. Further analysis revealed that DTX3 regulated CRC cell growth via regulating E2F transcription factor 1 (E2F1), a master regulator of cell cycle [13], and its downstream genes. These results support further preclinical evaluation of DTX3 as a therapeutic target for CRC.

## Materials and methods

### Gene expression profile analysis

The online databases Gene Expression Profile Interactive Analysis (GEPIA, http://gepia.cancer-pku.cn/index.html) [14] and Oncomine (https://www.oncomine.org/resource/login.html) [15] were used to assess the expression of DTX3 in colorectal tumors and normal colorectal tissues.

### Cell culture and transfection

Human-derived CRC cell lines (HCT116, RKO, SW480, DLD1) and 293T were original from ATCC. All cells were cultured in a humidified incubator with 5% CO_2_ at 37 °C. All CRC cells were maintained in McCoy’s 5 A medium (Hyclone, Logan, UT, USA); and 293T cells were cultivated in DMEM (Hyclone); DLD1 cells were maintained in RPMI-1640 medium (Hyclone). All culture medium was supplemented with 10% fetal bovine serum (FBS) (Hyclone), 100 mg/mL streptomycin, and 100 U/mL penicillin (Hyclone, Logan, UT, USA).

DTX3 overexpression constructs were generated by subcloning the PCR-amplified DTX3-coding sequence into lenti-virus vectors. To knock out endogenous *DTX3*, sgRNA sequences oligonucleotides targeting human *DTX3* were cloned into Lenti-V2 vectors. Upon reaching 70–80% confluence, cells were transiently transfected with vectors containing DTX3-coding sequence, sgRNA targeting DTX3, or corresponding empty vectors using Lipofectamine 2000 Transfection Reagent (Invitrogen, Carlsbad, CA). Stably transfected cell lines were screened by puromycin (Invitrogen) at 48 h after transfection. Transfection efficacy was evaluated by measuring the expression level of DTX3 in cells using Western blot.

### Serum starvation treatment

Cells subjected to serum starvation were cultured in serum-deprived culture medium for 24 h. Then cells were put back to fresh culture medium containing 10% FBS for 12 h. Total RNA was extracted for quantitative real-time PCR (qRT-PCR) analysis.

### *E2F1* inhibitor treatment

PD 0332991 is an inhibitor of cyclin-dependent kinases 4 and 6 and exhibits a potent inhibitory effect on the transcriptional activity of E2F1 [16]. Cells subjected to PD 0332991 (Selleckchem, TX, USA) administration were pre-treated with 20 nM of PD 0332991 for 12 h and then with 100 nM of PD 0332991 for additional 12 h. Control cell were treated with the same volume of Dimethyl Sulfoxide (DMSO) following the same procedure.

### Colony formation assay

Cells were pre-treated with 10 nM of PD 0332991 for 12 h and seeded into 6-well plates (4 × 10^2^/well) and maintained in a 5% CO_2_ incubator at 37 °C for 12 days. The colonies were then fixed with 4% paraformaldehyde and stained with 0.5% crystal violet. The number of colonies was counted under a light microscope.

### CCK-8 assay

Cells were plated into 96-well plates (1 × 10^3^/well) and cultured in a 5% CO_2_ incubator at 37 °C. At 1, 3, and 5 days after seeding, 100 µL of Cell Counting Kit-8 (CCK-8 solution) diluted in culture medium was added to each group of cells. After 90 min of incubation, the absorbance (optical density) at 450 nm was measured by a microplate reader FLx800 (BioTek, Vermont, USA).

### Dual-luciferase reporter assay

The promoter binding motif of the *E2F1* downstream genes was PCR-amplified and cloned into the firefly luciferase reporter vector. The 293T cells were co-transfected with 100 ng of firefly luciferase reporter vectors, 20 ng of Renilla luciferase expression plasmids, and plasmid vectors containing DTX3-coding sequence at different concentrations (0, 100, 200, 300, and 400 ng). HCT116 cells were infected with Lenti-V2 vectors containing sgRNA sequences targeting DTX3 or empty vectors. Then cells were co-transfected with 400 ng of firefly luciferase reporter vectors containing the promoter region of the *E2F1* gene and 20 ng of Renilla luciferase expression plasmids. Luciferase activity was measured at 48-h post-transfection using a Dual-Luciferase Reporter Assay System [17].

### RT-PCR

Total RNA was extracted from cells using TRIzol Extraction Kit (Invitrogen). cDNA was synthesized using PrimeScript RT Reagent Kit (Roche, Alameda, CA, USA). qRT-PCR analysis was performed using a TaqMan Probe Master Mix Kit (Takara, Tokyo, Japan). GAPDH was used as the internal control. The primers used for amplification are as follows [8]: Cdc2 forward: 5′-GGTCAAGTGGTAGCCATGAAA-3′, Cdc2 reverse: 5′-CCAGGAGGGATAGAATCCAA-3′; cyclin D3 forward: 5′-AGGGATCACTGGCACTGAAG-3′, cyclin D3 reverse: 5′-GGTGTATGGCTGTGACATCTG-3′; GAPDH forward: 5′-GAGTCAACGGATTTGGTCGT-3′, GAPDH reverse: 5′-GACAAGCTTCCCGTTCTCAG-3′.

### Western blot

Total protein was extracted from cells using lysis buffer (10 mM Tris–HCl pH 7.4, 150 mM NaCl, 0.5% NP40) containing protease inhibitors (Roche). Protein concentration in each sample was determined by the BCA assay. Equal amounts of protein (20 µg) were loaded on 10% SDS-PAGE gels and then transferred to polyvinylidene difluoride membranes (PVDF). The membranes were then incubated with the following primary antibodies overnight at 4 °C: DTX3 (25304-1-AP, Proteintech, Wuhan, China), CDC2 (#9116, CST, Danvers, MA, USA), E2F1 (#3742, CST), GAPDH (#5174, CST). After incubation with a secondary antibody for 1 h, the protein bands were visualized using an enhanced chemiluminescence system. The intensity of the bands was quantified using ImageJ.

### Statistical analysis

All experiments were performed in triplicate and repeated three times. Data were shown as mean ± standard deviation and analyzed using SPSS software (version 24.0). One-way analysis of variance (ANOVA) followed by Bonferroni post-hoc test was used to determine statistical significance. A *p*-value of less than 0.05 was considered statistically significant.

## Results

### DTX3 is downregulated in colorectal tumor tissues

**Fig. 1:**
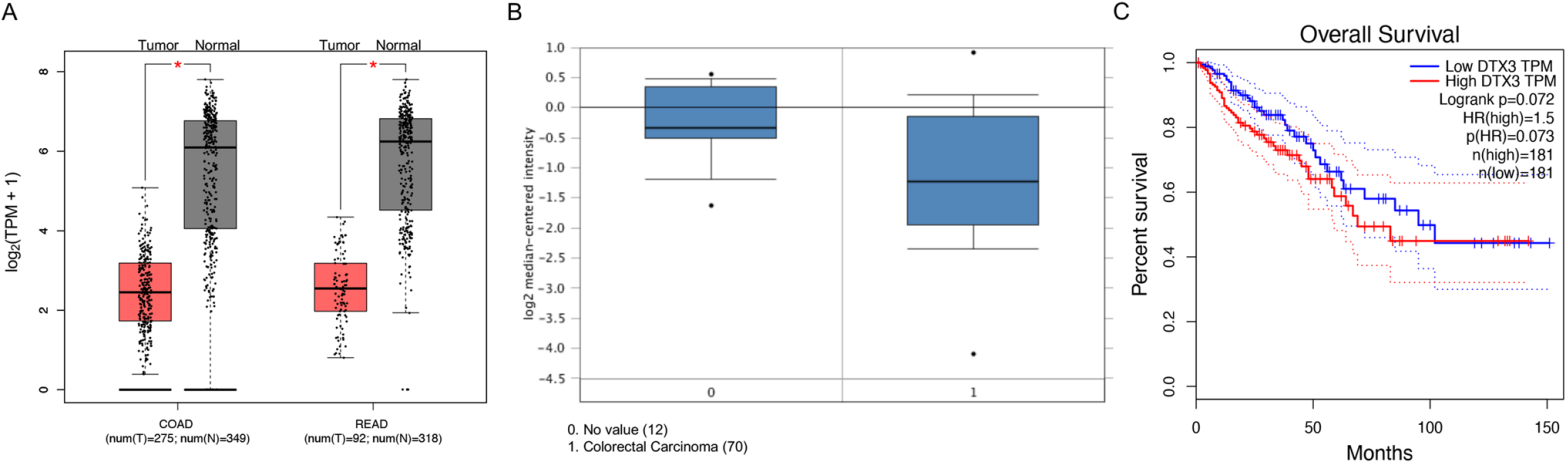
The expression of DTX3 in CRC and normal tissues. The gene expression profile of colorectal tumors and normal colorectal tissues was obtained from **A** GEPIA and **B** oncomine online databases. **C** The overall Kaplan–Meier analysis based on the expression of DTX3 in colorectal cancer tissues using the GEPIA website. The expression of DTX3 in tumor and normal tissues was compared. *COAD* colon adenocarcinoma, *READ* rectum adenocarcinoma. *p < 0.05

The expression of DTX3 in colorectal tumors and normal colorectal tissues was first assessed using the GEPIA database. A significant downregulation of DTX3 was observed in colon adenocarcinoma (n = 275) and rectum adenocarcinoma (n = 92) tissues in comparison with normal tissues (n = 349 and n = 318 respectively) (Fig. 1 A). Consistently, the data from the Oncomine database showed significantly reduced DTX3 expression in CRC tissues (n = 70) relative to normal tissue samples (n = 12) (Fig. 1B). In addition, Kaplan–Meier analysis showed that CRC patients with high DTX3 expression had poorer survival ratio (Fig. 1C). These findings suggested that the downregulation of DTX3 may be associated with the pathogenesis of CRC.

### The overexpression of DTX3 reduces colony-forming capacity and proliferation of CRC cells

**Fig. 2:**
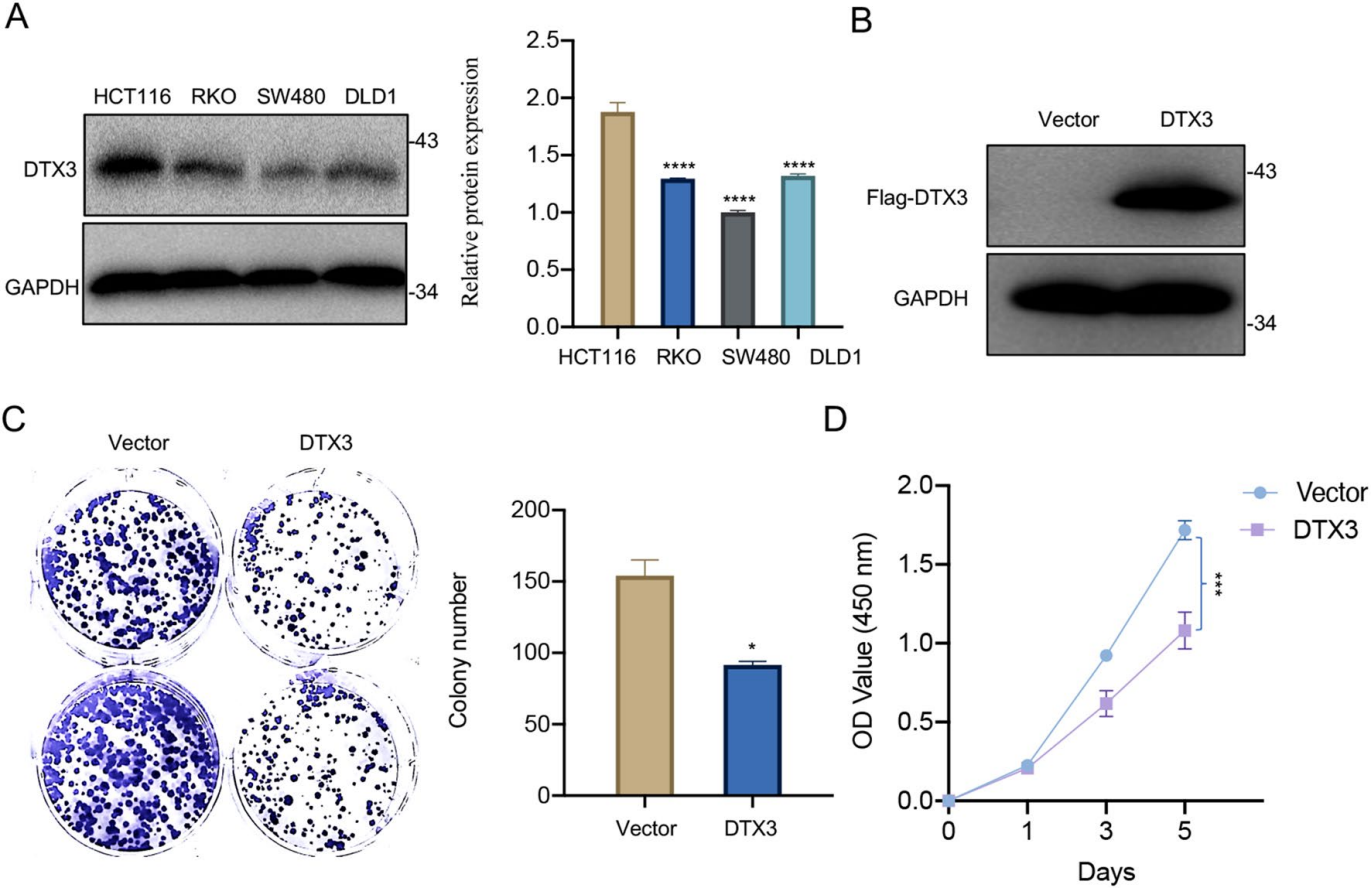
Effect of DTX3 overexpression on the colony-forming capacity and proliferation of CRC cells. **A** The protein expression of DTX3 in four CRC cell lines was determined by Western blot and normalized to that of GAPDH. **B**–**D** RKO cells were transfected with plasmid vectors containing DTX3-coding sequence (DTX3) or empty vectors (Vector). **B** The cellular expression of DTX3 at 48-h post-transfection was assessed by Western blot. **C** The colony formation assay was performed to evaluate the colony-forming capacity of RKO cells. After 12 days, the number of colonies was counted. **D** The CCK-8 assay was used to assess the proliferation of cells. The optical density (OD) value was recorded at 1, 3, and 5 days after seeding

To determine whether DTX3 plays a regulatory role in CRC progression, we first measured the expression of DTX3 in different CRC cell lines. The highest protein level of DTX3 was observed in HCT116 cells relative to other cell lines (RKO, SW480, and DLD1) (Fig. 2A). Therefore, HCT116 cells were used to investigate the effect of DTX3 KO, while RKO cells were selected to analyze the effect of DTX3 overexpression in subsequent experiments. RKO cells transfected with vectors containing DTX3-coding sequence showed markedly increased expression of DTX3 compared with those transfected with empty vectors (Fig. 2B). The overexpression of DTX3 significantly reduced the colony-forming capacity of RKO cells (Fig. 2C). Cells with upregulated DTX3 expression also showed significantly decreased proliferation, as evidenced by lower OD value compared with the empty vector-transfected group (Fig. 2D). The above results suggested that DTX3 overexpression reduced colony-forming capacity and proliferation of CRC cells.

### The knockout of DTX3 promotes CRC cell growth

**Fig. 3:**
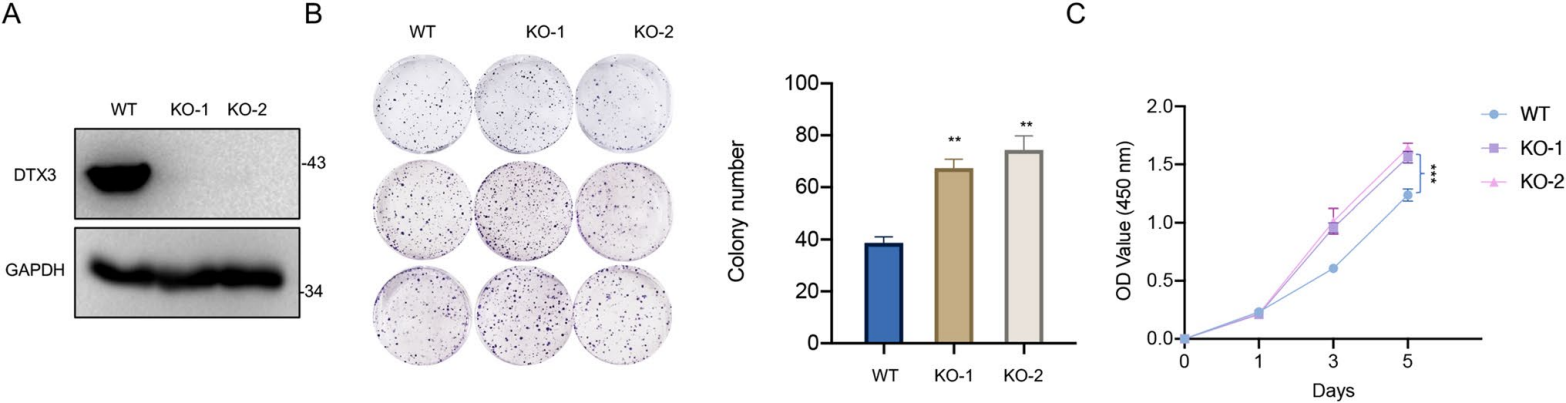
Effect of DTX3 knockout on the colony-forming capacity and proliferation of CRC cells. HCT116 cells were transfected with vectors containing sgRNA sequences targeting DTX3 (KO-1 and KO-2) or empty vectors (WT). **A** The protein expression of DTX3 at 48-h post-transfection was measured by Western blot. **B** The colony formation assay was performed to evaluate the colony-forming capacity of HCT116 cells. After 12 days, the number of colonies was counted. The number of colonies in each group was counted. **C** The CCK-8 assay was performed to assess the proliferation of transfected cells. The optical density (OD) value was recorded at 1, 3, and 5 days after seeding

To investigate the effect of DTX3 KO on CRC cells, we transfected HCT116 cells with vectors containing sgRNA sequences targeting DTX3 (KO-1 and KO-2) or empty vectors. Western blot analysis showed that DTX3 was knocked out following the transfection with sgRNA vectors (Fig. 3A). We further analyzed the colony-forming capacity and proliferation of HCT116 cells following transfection. The results revealed that DTX3 KO significantly promoted the (Fig. 3B) colony formation and (Fig. 3C) proliferation of CRC cells, indicating that the absence of DTX3 is essential for CRC progression.

### DTX3 regulates CRC cell growth via affecting the E2F1 and transcription of its downstream genes

**Fig. 4:**
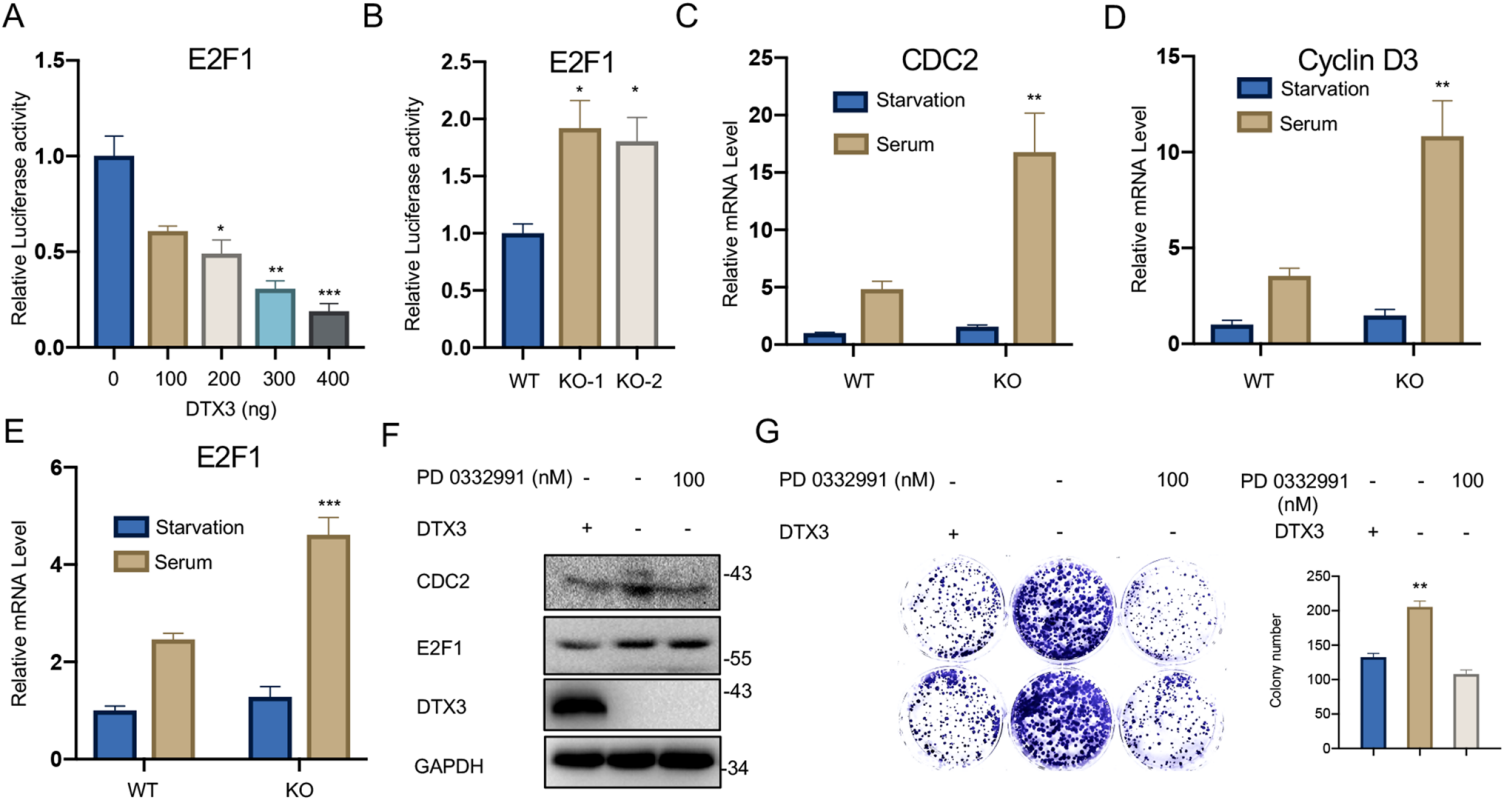
Effect of DTX3 knockout on the transcription of E2F1 and its downstream genes in CRC cells. **A** The 293T cells were co-transfected with firefly luciferase reporter vectors, Renilla luciferase expression plasmids, and vectors containing DTX3-coding sequence at various concentrations. Luciferase activity was measured at 48-h post-transfection. **B** HCT116 cells were transfected with vectors containing sgRNA sequences targeting DTX3 or empty vectors. Then cells were co-transfected with firefly luciferase reporter vectors containing the promoter region of the *E2F1* gene and Renilla luciferase expression plasmids. Luciferase activity was measured at 48-h post-transfection. **C**–**E** HCT116 cells were transfected with vectors containing sgRNA sequences targeting DTX3 (KO) or empty vectors (WT). Then cells subjected to serum starvation were cultured in serum-deprived medium for 24 h, followed by incubation in fresh medium containing 10% FBS for 12 h. The “Serum” group was maintained in culture medium containing 10% FBS during the experiment. Then total RNA was extracted from cells. The mRNA levels of **C** E2F1, **D** CDC2 and **E** Cyclin D3 were measured by qRT-PCR. **F**, **G** HCT116 cells were transfected with vectors containing sgRNA sequences targeting DTX3 (DTX3–) or empty vectors (DTX3+). Cells subjected to PD 0332991 administration (PD 0332991+) were pre-treated with 20 nM of PD 0332991 for 12 h and then with 100 nM of PD 0332991 for additional 12 h. The other groups (PD 0332991–) were treated with the vehicle following the same procedure. **F** The protein expressions of CDC2, E2F1, and DTX3 were measured by Western blot. **G** The colony formation assay was performed to evaluate the colony-forming capacity of HCT116 cells in the presence or absence of PD0332991. After 12 days, the number of colonies was counted

To further elucidate the mechanisms underlying DTX3-mediated CRC cell growth, we performed dual-luciferase reporter assay to examine the effect of DTX3 on the transcriptional activity of the E2F1 promoter. We found that the transcriptional activity of E2F1 was significantly and concentration-dependently decreased with the increase in the DTX3 level (Fig. 4 A). However, the silence of DTX3 notably enhanced the transcriptional activity of E2F1 (Fig. 4B). Next, we examined the mRNA levels of E2F1 downstream genes, CDC2 and Cyclin D3, in transfected CRC cells cultured in serum-deprived or normal culture medium. We found that the expressions of CDC2 and Cyclin D3 remained low in both WT and KO cells under starved conditions. When cultured in serum-containing medium, however, DTX3 deletion significantly elevated the mRNA levels of E2F1, CDC2 and Cyclin D3 in CRC cells (Fig. 4C–E). To ascertain that DTX3 affected the expression of E2F1 in CRC cells, we treated DTX3 KO cells with or without PD 0332991, an agent that shows a potent inhibitory effect on the transcription of E2F1. The results demonstrated that DTX3 KO upregulated the protein levels of E2F1 and its downstream target CDC2 in HCT116 cells, while the addition of PD 0332991 diminished the regulatory effect of DTX3 KO on CDC2 expression (Fig. 4F). In addition, treatment with PD 0332991 suppressed DTX3 KO-induced colony formation in CRC cells (Fig. 4G). Taken together, these data indicated that DTX3 regulated CRC cell growth via affecting E2F1.

## Discussion

Pharmacological molecules that selectively target the genes encoding cell cycle-related proteins provide an insightful perspective in CRC treatment [18]. Here, we reported that DTX3 inhibited CRC cell proliferation and regulated the expression of cell cycle regulators via effecting E2F1. These findings highlighted the potential of DTX3 as a therapeutic target in CRC.

DTX3 is an understudied E3 ubiquitin ligase that has been reported to regulate the ubiquitination and stabilization of endogenous proteins in cancer cells. High ectopic expression of DTX3 promoted ovarian cancer cell proliferation and invasion by stabilizing mutant p53 and inducing the expression of its target genes [11]. However, DTX3 plays a contradictory role in the progression of esophageal cancer, during which it suppresses tumorigenesis by promoting the ubiquitination and degradation of NOTCH2, a key protein implicated in cell proliferation and cell-fate determination [12]. The anti-metastatic effect of DTX3 attributed to its ubiquitin ligase activity has also been reported in triple-negative breast cancer [19]. In the current study, we observed a significant downregulation of DTX3 in CRC tissues relative to normal colorectal tissues. Moreover, DTX3 overexpression inhibited, while DTX3 KO promoted the colony-forming capacity and proliferation of CRC cells, suggesting that insufficient DTX3 expression was a prerequisite for CRC progression.

E2F1 is a key cell-cycle mediator that participates in the progression, metastasis, and chemoresistance of CRC [20]. The in vitro data showed that E2F was overexpressed in CRC cells and promoted the resistance of cells to 5-fluorouracil, a drug widely used for treating CRC [21]. Moreover, clinical studies have associated E2F1 with poor prognosis in CRC patients [22]. In the current study, we found that the transcriptional activity of E2F1 was significantly reduced with the increase in the cellular level of DTX3, while DTX3 KO exerted an opposite effect. Previous evidence has revealed that the stability of E2F1 can be regulated by the proteasome system via ubiquitination, resulting in dysregulated cell cycle progression [23]. Wang et al. found that downregulation of ubiquitin-specific protease 11 increased E2F1 ubiquitination and reduced E2F1 stability, thereby suppressing cell proliferation and wound healing in lung epithelial cells [24]. Zhou et al. reported that GSK3beta promoted the ubiquitination of E2F1 and thus inhibited its transcription activity during neural cell differentiation [25]. The transcription activity of E2F1 may due to enhanced E2F1 promoter binding ability or upregulated E2F1 level. Our results shows enhanced E2F1 protein level is an important part of upregulated E2F1 transcription activity. However, considering the ubiquitin ligase activity of DTX3 directly targeting NOTCH2 in esophageal cancer and breast cancer [12, 19], whether DTX3 regulates the transcriptional activity of E2F1 via ubiquitination or interacting with other factors indirectly activating E2F1 pathway warrants further investigation.

We further examined the expression of E2F1 target genes related to cell cycle progression, CDC2 and Cyclin D3. CDC2 not only induces spontaneous proliferation of cancer cells but also leads to rapid tumor growth [26]. The upregulation of CDC2 is associated with increased risk of distant metastasis and reduced survival time in CRC [27, 28]. Cyclin D3 controls cell cycle progression and has been recognized as an adverse predictor in multiple human malignant neoplasms, including CRC [29]. The retinoblastoma protein as an inhibitor of E2F1 control the transcriptional activity of E2F1. PD0332991 blocks the retinoblastoma protein from being phosphorylated and thus affects E2F1 activity. Here, our results demonstrated that DTX3 KO significantly elevated, while the addition of PD 0332991, an inhibitor of E2F1 transcription, inhibited the expression of both CDC2 and Cyclin D3 in CRC cells. Additionally, treatment with PD 0332991 suppressed DTX3 KO-induced colony formation in CRC cells. These findings need to be validated in vivo.

In conclusion, DTX3 plays an indispensable role in promoting tumor cell growth and mediating cell cycle-related proteins in CRC. Our study provided a rationale for developing therapeutic methods targeting the DTX3/E2F1 axis for CRC patients.

## Data Availability

The datasets used or analyzed during the current study are available from the corresponding author upon reasonable request.
